# Towards a reporting guideline for developmental and reproductive toxicology testing in *C. elegans* and other nematodes

**DOI:** 10.1093/toxres/tfab109

**Published:** 2021-11-28

**Authors:** Monique van der Voet, Marc Teunis, Johanna Louter-van de Haar, Nienke Stigter, Diksha Bhalla, Martijn Rooseboom, Kimberley E Wever, Cyrille Krul, Raymond Pieters, Marjolein Wildwater, Vera van Noort

**Affiliations:** Vivaltes, 1704 NA, Heerhugowaard the Netherlands; Utrecht University of Applied Sciences, Innovative testing in Life Sciences & Chemistry, 3584 CH, Utrecht, the Netherlands; Utrecht University of Applied Sciences, Innovative testing in Life Sciences & Chemistry, 3584 CH, Utrecht, the Netherlands; Utrecht University of Applied Sciences, Innovative testing in Life Sciences & Chemistry, 3584 CH, Utrecht, the Netherlands; KU Leuven, Centre of Microbial and Plant Genetics, Faculty of Bioscience Engineering, 3001, Leuven, Belgium; Toxicology group Shell International B.V., 2596 HR, The Hague, the Netherlands; Radboud University Medical Center, Radboud Institute for Health Sciences, Department for Health Evidence, 6525 GA, Nijmegen, the Netherlands; Utrecht University of Applied Sciences, Innovative testing in Life Sciences & Chemistry, 3584 CH, Utrecht, the Netherlands; Utrecht University of Applied Sciences, Innovative testing in Life Sciences & Chemistry, 3584 CH, Utrecht, the Netherlands; Utrecht University, Institute for Risk Assessment Sciences, 3584 CM, Utrecht, the Netherlands; Vivaltes, 1704 NA, Heerhugowaard the Netherlands; Utrecht University of Applied Sciences, Innovative testing in Life Sciences & Chemistry, 3584 CH, Utrecht, the Netherlands; KU Leuven, Centre of Microbial and Plant Genetics, Faculty of Bioscience Engineering, 3001, Leuven, Belgium; Leiden University, Institute of Biology Leiden, 2333 BE, Leiden, the Netherlands

**Keywords:** reduction, refinement and replacement (3Rs), new approach methodologies (NAMs), developmental and reproductive toxicology (DART)

## Abstract

Implementation of reliable methodologies allowing Reduction, Refinement, and Replacement (3Rs) of animal testing is a process that takes several decades and is still not complete. Reliable methods are essential for regulatory hazard assessment of chemicals where differences in test protocol can influence the test outcomes and thus affect the confidence in the predictive value of the organisms used as an alternative for mammals. Although test guidelines are common for mammalian studies, they are scarce for non-vertebrate organisms that would allow for the 3Rs of animal testing.

Here, we present a set of 30 reporting criteria as the basis for such a guideline for Developmental and Reproductive Toxicology (DART) testing in the nematode *Caenorhabditis elegans*. Small organisms like *C. elegans* are upcoming in new approach methodologies for hazard assessment; thus, reliable and robust test protocols are urgently needed. A literature assessment of the fulfilment of the reporting criteria demonstrates that although studies describe methodological details, essential information such as compound purity and lot/batch number or type of container is often not reported. The formulated set of reporting criteria for *C. elegans* testing can be used by (i) researchers to describe essential experimental details (ii) data scientists that aggregate information to assess data quality and include data in aggregated databases (iii) regulators to assess study data for inclusion in regulatory hazard assessment of chemicals.

## Introduction

Developmental and Reproductive Toxicity (DART) is a critical safety evaluation of chemicals under chemical legislations throughout the world. In the European context, chemicals are controlled under REACH (Registration, Evaluation and Authorization of Chemicals). The goal of REACH is to protect human health and the environment. In the EU, a chemical compound cannot be used or imported until sufficient information has been provided regarding the safety of the compound [1]. In case of higher volumes put on the market, more information needs to be provided. ECHA (the European Chemicals Agency) critically evaluate the results of safety tests and maintain the ECHA database [2]. In the USA, the National Toxicology Program (NTP) provides information on toxicity and human health risks of compounds. Information is stored in the United States Environmental Protection Agency (US-EPA) Toxicity Reference Database (ToxRefDB) [3].

Current safety testing of chemicals is typically carried out according to guidelines established by the Organisation for Economic Cooperation and Development (OECD). These guidelines include instructions on how to carry out safety testing in rodent and non-rodent animal models, as well as in *in vitro* tests, e.g. skin and eye irritation tests. For evaluating DART, the OECD guidelines describe pre-, post-, and perinatal development, and multiple generation testing in rodents and non-rodent mammals (Table 1). For example, OECD test guideline 415 describes how tests for effects on the male and female reproductive system should be carried out. A minimal number of pregnant animals are required, as well as daily observations of the animals. As a second example, guideline 414 describes testing for prenatal developmental toxicity and is intended for use with pregnant rats and rabbits. Foetuses are to be examined for abnormalities after caesarean section.

**Table 1:** OECD guidelines for DART

Test guideline	Title
OECD TG 408	Repeated Dose 90-day Oral Toxicity Study in Rodents
OECD TG 414	Prenatal Developmental Toxicity Study
OECD TG 415	One-Generation Reproduction Toxicity Study
OECD TG 416	Two-Generation Reproduction Toxicity Study
OECD TG 421	Reproduction Developmental Toxicity Screening Test
OECD TG 422	Combined 28-Day Repeated Dose Toxicity Study with the Reproduction/Developmental Toxicity Screening Test
OECD TG 426	Developmental Neurotoxicity Study
OECD TG 433	Extended One-Generation Reproductive Toxicity Study

There are drivers to Replace, Reduce, and Refine testing with vertebrate animal models (3Rs), the come from the public, governmental authorities, the scientific community, and industry. Besides ethical reasons, data from animal tests may have limited translational value for humans health [4, 5]. Other factors may also play a role in moving to alternative methods: animal testing is costly, labour, and resource intensive and there are limitations in the number of tests that can be performed due to resource limitations at test facilities [6]. In response to these incentives, it has been shown that non-mammalian model species may provide valuable information concerning DART [7–9] especially if the specific class of compound is considered.

New Approach Methodologies (NAMs) can provide a first decisive tier in hazard assessment through DART screening of compounds, after which the safety of only a limited set of compounds is further investigated by vertebrate tests [9]. Promising examples of these NAMs for DART are the nematode *Caenorhabditis elegans*, the zebrafish embryo *Danio rerio*, and the slime mold *Dictyostelium discoideum*. However, no standardized protocols currently exist describing how DART tests should be carried out in NAMs and which quality criteria should be fulfilled to obtain a reliable result. Reporting guidelines describing what experimental information should be reported for DART in order to independently judge the quality of a study are also lacking.

Guidelines related to NAM species that are not specific to DART are OECD 212 and 236, describing short-term toxicity and acute toxicity in fish embryos to assess aquatic toxicity. In addition, ISO 10872 describes tests for toxic effects of sediment and soil samples on growth, fertility, and reproduction of *C. elegans*. Beronius et al. [10] have initiated evaluation and reporting criteria intended for researchers performing *in vivo* toxicity studies, including DART. However, these criteria cannot be readily applied to tests using *C. elegans*, since these were formulated with vertebrates in mind. For example, *C. elegans* nematodes are not easily identified individually, and bedding and water bottle conditions do not apply to *C. elegans* tests. Guidelines that do take NAM species into account are the ARRIVE 2.0 guideline (Animal Research: Reporting of *In Vivo* Experiments), updated by the UK National Centre for the 3Rs (NC3Rs). These guidelines describe publication considerations for animal research, including mammalian species and model organisms such as *Drosophila melanogaster* and *C. elegans* [11]*.* Specific *C. elegans* guidelines for reliable results in toxicity testing by good *C. elegans* culture practice (GCeCP) are described by Hunt [12].

This in-depth review aims to (i) get insight into the representation of NAM species in recent publications of DART tests (ii) formulate a reporting guideline to assess DART specifically in *C. elegans*, and (iii) assess the reporting quality of DART studies based on those guidelines.

The quality criteria were formulated starting from the SciRAP criteria developed by Beronius et al. (2014). We then performed a comprehensive literature search, screened abstracts for inclusion, and assessed the reporting quality of eligible publications according to the pre-set criteria. Most studies fulfilled the majority of criteria, and some criteria are fulfilled by all studies. We conclude that there is substantial consensus in the field on what methodological details are important to report, which is an essential first step towards developing a future *C. elegans* DART test guideline.

## Materials and Methods

### Literature mining

The complete search strategy is presented in Supplementary Table 1. We queried Medline (via Pubmed) on 20 February 2020 for studies on DART in six different species: rat, rabbit, mouse, zebrafish, *C. elegans,* and *D. discoideum*. We limited our search to records published after January 2018 to obtain the most current practice. We analysed the *C. elegans* studies in-depth, by manually pre-screening records related to *C. elegans* based on the title and abstract and, if deemed relevant, further screening based on the full text. The criteria to exclude the study based on the abstract were (i) no DART outcomes reported (ii) no chemical exposure data, (iii) not species of interest, and (iv) not a primary study.

The full text of the study was included in the review if it met the following inclusion criteria: (i) DART outcomes reported (ii) chemical exposure data (iii) species of interest and (iv) wild-type animals.

Two readers each read half of the full-text articles.

### New guideline formulation

A team of interdisciplinary experts was assembled to formulate a reporting guideline for *C. elegans* experiments optimized for guideline acceptance and data processing. The team includes (i) experts in in the field of *C. elegans* research (MV, MW, JL, NS, RP), (ii) experts in the field of OECD guideline studies and risk assessment in the field of registration studies for pharmaceuticals and chemicals (MR), OECD guideline studies for regulatory *in vitro* (geno)toxicity testing for the registration of chemicals, food ingredients, and pharmaceutical products (CK), and formal validation and pre-validation studies for inclusion of tests in the OECD, related to ECVAM activities (MT), and (iii) experts in the field of data science and bioinformatics (MV, VN, DB, MT, KW), with experience in meta-research, including comprehensive searching. The TIER-I and -II criteria from SciRAP were the basis for formulating the *C. elegans* criteria. These criteria were adjusted and amended to fit the needs of studies in *C. elegans*.

### Scoring

All full-text articles were scored for the presence of details on each of the 30 reliability criteria (Table 2). The details were logged in a spreadsheet. If the details were incomplete, it was scored as false. If the information could be inferred from the context, it was scored as true. Both the total number of studies reporting each criterion was counted and the number of reported criteria per study.

**Table 2:** Reporting guideline for DART testing in *C. elegans* and other nematodes

Criteria	Explanation	Fulfilled %
Compound		
Identifier	Compound name, ID, or CAS-number should allow for the unambiguous identification of the compound.	100
Source	The compound composition can vary by source, thus report the manufacturer and batch/lot number.	9
Purity	Compound purity is important to consider; information on contaminants and isomers should be traceable based on the manufacturer and lot/batch number or reported by the researcher.	48
Vehicle/solvent		
Type/characteristics	The type of vehicle or solvent is important in relation to the compound being studied, e.g. a very hydrophobic compound will not dissolve in water. An example is a study that found that the fungicide pyraclostrobin is not dissolved in two vehicles used in industry-sponsored toxicity studies [37]. Thus, report the type and characteristics of the vehicle or solvent.	91
Animals		
Species	This guideline is developed based on *C. elegans* expertise but applies to other nematode species such as *Caenorhabditis briggsae* (for a list of nematode species with an established research community, see https://wormbase.org/species/).	100
Strain	Reporting the complete genetic nomenclature is important for understanding background elements that might influence the phenotype [38, 39].	96
	Give information on experimental animals and controls and whether strains were outcrossed to remove background mutations that can accumulate over time. Unambiguous identity identification is recommended, for example, by sequencing, to make sure no contamination of the stock has taken place. To prevent genetic drift, it is recommended to work from frozen stocks and track generation time.	
Source	Provide a reference to the source of the animals, for example, Caenorhabditis Genetics Center (CGC; https://cgc.umn.edu/), publications, or lab information.	83
Sex	Explicitly state whether hermaphrodites or males were used in specific experiments. Sex-specific toxicity responses have been observed during *C. elegans* development [40]. In addition, reproductive rates vary widely between mixed and pure hermaphrodite populations.	39
Culture conditions during the administration of the compound		
Temperature (°C)	*C. elegans* can be grown at temperatures ranging from 15–25°C; small changes can impact developmental timing [41], reproduction [41], metabolism [23], and lifespan [42].	91
Method to maintain quality of media	Agar plates dry out at room temperature, losing ∼2% of their water per day [17]. Evaporation can cause solid and liquid media to become more concentrated, especially when using small volumes. Report how media quality is maintained, e.g. by using fresh media or controlling ambient humidity.	0
Light–dark cycle	It is not common to provide information on the lighting conditions of *C. elegans* cultures. It is important to include this information in light of insights that circadian rhythms influence metabolic variables [43] and nematodes are sensitive to visible light, which reduces longevity [44].	13
Container type	The type of container (e.g. open or closed) is important to report, especially when handling compounds with volatile properties that can evaporate, lowering the exposure.	9
Container material	The material of the container (e.g. plastic or glass) can impact the research outcome. A hydrophobic compound, for example, can stick to plastic and the freely available concentration will be reduced [15, 16].	13
Media composition during exposure	There are various ways of culturing nematodes; this can be performed in solid agar or liquid medium. The uptake of the compound (and thus the exposure) depends on many variables and can vary based on the media used [14]. For example: is the compound mixed through the agar medium or applied on top. The solution pH of liquid medium should be measured and buffered with appropriated buffers as acidity can affect nematode survival [18, 19].	96
Food Type and source	Nematodes are commonly fed with a bacterial food source; this creates the confounding problem of the metabolic response of the feeder organism. A live culture may create toxic compounds (e.g. reactive oxygen species) under certain metabolic conditions [21]. Killing bacteria through UV, heat, or antibiotics can still impact experimental outcome [22]. The bacterial species used is also known to affect *C. elegans* metabolism [23]. Carefully describe the food type: axenic or bacterial and the bacterial handling method if applicable (e.g. inactivation method). To rule out batch effects, it is recommended to compare controls and establish baseline values consistent from batch to batch.	96
Food amount	Not just the food type and source are important, but also the amount added to the assay. Live bacteria can metabolize compounds; in addition, the bacteria can adsorb compounds on their surface, thus changing the exposure [24].	22
Administration of compound		
Administration method	Is the compound delivered through spiking or passive dosing, e.g. using rings/discs? The administration method influences the freely dissolved chemical concentration and thereby the toxic response [45].	100
Agitation method for liquid medium	Nematodes cultured in a liquid medium need to be agitated to aerate the substrate to grow the nematode and symbiotic bacteria. The method of agitation influences the endpoint, for example, by protein denaturation [16]. Report the type of agitation (e.g. shaking or rolling) and the rotations per minute (rpm).	70
Number of animals/container when exposed to the compound	A high density of nematodes will reduce nutrient availability and metabolize the compound to which they are exposed more quickly. In addition, secreted hormones can influence the development of the population [46].	57
Number of animals/sex/dose group	The sample size (n) is important to assess the robustness of the experimental setup and the chosen statistical method.	70
Dose levels or concentrations and number of dose groups	Adequate information is needed to plot a dose–response curve and extract parameters such as EC50, IC50, ED50.	100
Frequency of administration	Various administration methods are possible; report whether the dose was administered once, repeated, or continuous.	100
Duration of administration	Report the duration and timeframe during which the administration took place, e.g. hours, days, or age of the nematode.	96
Age and life stage of animals at the start of administration	To interpret developmental and reproductive toxicity, it is important to report the life stage at which the compound was first administered, e.g. during the parental L1 stage, L4 stage, or adults; or directly in the experimental population. Toxicity can be life-stage dependent [47].	91
The number of replicates per dose level/concentration or the number of times the experiment was repeated	The number of replicates is important to assess the robustness of the experimental setup and the chosen statistical method.	70
Information about controls	Report information on both the negative control (e.g. vehicle or solvent) and positive control (e.g. compound with known effect in the assay). Historical controls (data from past studies) are discouraged unless compared with current controls to control for batch effects.	83
Examinations		
Details of examinations including all adverse events in each experimental group	Report in detail the observations that were made during the experiment, including presence of Dauer stage nematodes as these can reduce growth, delayed development in controls and dosed populations, population composition in terms of life stages and sex, and the method of measuring these parameters.	100
Analysis		
Response data by treatment group	Quantitatively report the data by treatment group.	100
Details of statistical methods applied	Report on the power calculation, sample size, researcher blinding, analysis method, adjustment for multiple comparisons.	96
Disclosure		
Disclosure of any potential conflicts of interest	Disclosure ensures a transparent publication process where the objective representation of data can be reviewed.	65

Table 2 list of 30 reporting criteria for a C. elegans DART test.

Criteria are listed along with an explanation and how often papers in our review fulfilled the criterion.

## Results and Discussion

**Figure 1:**
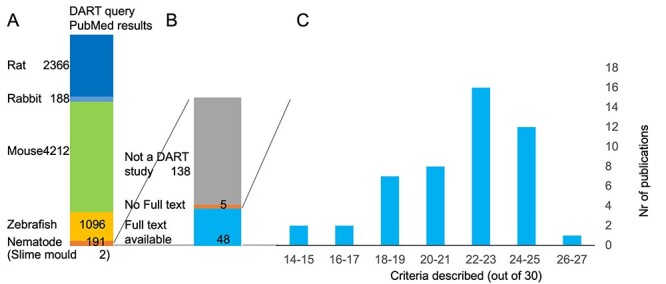
Overview of the review. (A) Number of resulting abstracts for each DART query of the six organisms. (B) Selection of abstracts of nematode DART studies. (C) Number of criteria described for full-text nematode DART studies.

Traditional species for toxicity studies are mouse, rat, and rabbit, but NAM species are gaining interest in the light of 3Rs. To get an overview of the contribution of NAM to the current literature, we performed a PubMed search for studies related to DART in six different species published after January 2018. We found 4212 publications for mouse, 2366 publications for rat, 188 publications for rabbit, 1096 publications for zebrafish, 2 publications for *D. discoideum,* and 191 publications for *C. elegans*. This suggests that most of the recent literature is focused on established model organisms such as rats and still relatively few studies are based on non-mammalian model organisms (Fig. 1A), here still lies an opportunity for 3Rs.

**Figure 2:**
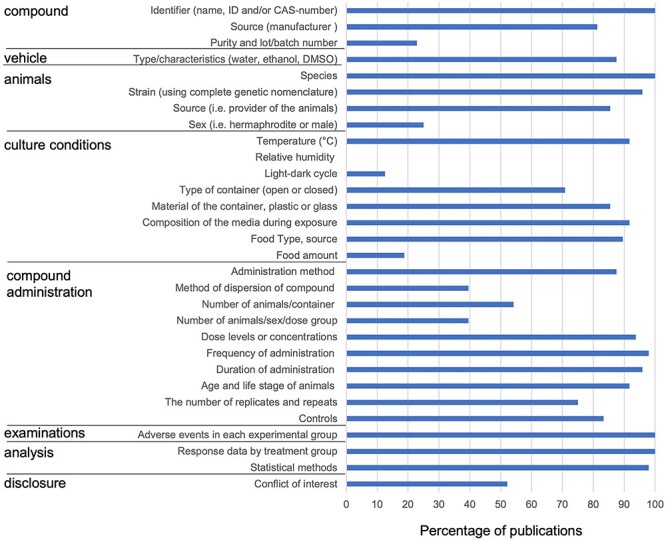
Percentage of analysed studies that report each of the proposed criteria.

To gain more insight into the reporting standards of recent *C. elegans* literature, we analysed the 191 *C. elegans* publications in more detail*.* The abstracts were pre-screened for relevance based on whether they contained DART outcomes, chemical exposure data, were experimentally performed in *C. elegans,* and were carried out with wild-type animals to guarantee a standardized animal population. This resulted in 53 (out of 191) publications to assess reporting quality based on the full text (Fig. 1B). For five publications, full-text articles were not accessible. To assess reporting quality, we formulated guidelines relevant to *C. elegans* (Fig. 2, Table 2). The SciRAP reporting checklist was used as a starting point (http://www.scirap.org/ downloaded 24 May 2017) [13], because the guidelines have a broad acceptance base: they are refined by scientists and professionals from authorities, academia, and industry with expertise in toxicology and risk assessment [10]. We adapted these guidelines with our team of experts to be relevant for *C. elegans* studies (Table 2). This involved discussions with experts in *C. elegans* lab testing, OECD guideline studies, and data analytics. Examples of SciRAP *in vivo* toxicity criteria that were not considered relevant are ethical review permissions, licences, and national or institutional guidelines for animal care and use, both not required for *C. elegan*s. Other criteria that were not relevant are body weight at the start of the study and the method for individual of animals.

We formulated new criteria that are critical in evaluating the quality of the assay that is being performed: the composition of the media during exposure; there are various ways of culturing nematodes; this can be performed in solid agar or liquid medium. The uptake of the compound (and thus the exposure) depends on many variables and can vary based on the media used [14]. For example, the compound can be mixed with the agar or added in a solution on top of the agar. The type of container is important; an open container may result in a reduced exposure when working with compounds with volatile properties (for example toluene). The material of the container may influence the test outcome; a hydrophobic compound can stick to the plastic container and thus lower the exposure creating false-negative results [15, 16]. The method to maintain the quality of media is important, e.g. by using fresh media or controlling ambient humidity; agar plates dry out at room temperature and can lose ca 2% of the water per day [17]. Evaporation can cause solid and liquid media to become more concentrated, especially when using small volumes. Fresh plates will also prevent nematode burrowing behaviour. The agitation method is important for liquid medium; nematodes cultured in a liquid medium need to be agitated to aerate the substrate to grow the nematode and symbiotic bacteria. The method of agitation influences the endpoint, for example, by protein denaturation [16]. The agitation type should be reported, for example shaking or rolling, including the rotations per minute (rpm). In liquid medium, the pH can also be affected. The toxicity can sometimes be ascribed to the pH rather than the specific compound [18]. In the case of basic or acidic compounds tested in liquid medium, alternate buffers should be used [19]. To assess the robustness of the experimental setup and the chosen statistical method it is important to report the number of replicates per dose level/concentration or the number of times the experiment was repeated.

Some criteria were rephrased, such as: ‘*administration method, e.g., if oral: via feed, gavage, drink from pipette, etc*’ to ‘*administration method, e.g., passive dosing rings/discs*.’ Some criteria were unchanged by do have special applications in C. elegans, for example food type and source. The food source is usually E. coli bacteria or axenic medium for nematodes; it should be described whether the bacteria are alive or dead and how much food was provided. This can influence study outcomes through the metabolization of compounds during exposure by bacteria [20], live cultures may create toxic compounds such as reactive oxygen species under certain metabolic conditions [21], killing bacteria through UV, heat, or antibiotics can also impact experimental outcome [22], the bacterial species used is known to affect C. elegans metabolism [23], and bacteria can adsorb compounds on their surface, thus changing the exposure [24]. It is important to carefully describe the food type: axenic or bacterial and the bacterial handling method if applicable (e.g. inactivation method). To rule out batch effects, it is recommended to compare controls and establish baseline values consistently from batch to batch. A detailed description of each formulated reporting guideline can be found in Table 2.

We scored the 48 *C. elegans* DART publications whether they reported each of the criteria in the different categories (Table 2, Fig. 2). The percentage of *C. elegans* DART studies that reported on the criteria are listed in Table 2. All studies reported the name of the compound used (a selection criterion), but the manufacturer of the compound was missing in one fifth of the publications (81.2%, 39/48). Purity and batch number are rarely reported (22.9%, 11/48) and can be a source for differences in test outcomes. For the purpose of reproducibility, both source and purity of a compound should be reported. The vehicle (water, DMSO, etc) was almost always mentioned (87%, 42/48). Information about the animals (species, strain, and source) was usually available (100% 48/48, 96% 46/48, 85% 41/48, respectively), although only a quarter of the studies reported the sex of the animals (25% 12/48), i.e. male or hermaphrodites. These details about sex and the way sex was determined are vital as sex-specific responses have been observed during *C. elegans* development. Moreover, the inclusion of males instead of hermaphrodites reduces growth rates as males are smaller and increase reproductive output. Details about the administration of the compound include frequency of administration (98% 47/48), duration of administration (96% 46/48), the age and life stage of animals at the start of administration such as L1 or L4 (92% 44/48).

For culturing conditions, most studies missed a lot of information. Generally, temperature (92% 44/48), composition of the media (92% 44/48) and food type are reported (90% 43/48). However, information on the amount of food (19% 9/48) as well as information on the state of the food (alive or dead) was often missing (Fig. 2). Describing the food status and quality is also of importance. *E. coli* bacteria are often fed to the nematodes, and living bacteria can take up the test compound and metabolize it [20], thereby affecting study results. However, in 17 out of 48 reports, we concluded that the food was likely alive from the method description. In addition, in 30 out of 48 cases we inferred whether the containers were open or closed. This information is essential as test outcomes can be affected when compounds with differences in physicochemical properties are not exposed suitably. Volatile compounds might evaporate so it is crucial to mention if containers were open or closed. An example of a hard-to-test compound is toluene, a volatile organic compound that can, for example, be present in gasoline [25]. The route of exposure, such as inhalation or skin exposure is very important for the observed toxic effects in humans [26]. For toluene, airborne exposure of nematodes via application to filter paper in a glass chamber as described in the reviewed article by Soares *et al.* [27], may have very different toxic effects than applying the compound in solution or mixing it with the agar plates. The latter is for example applied in a recent DART study using fruit flies where exposure to toluene is established by adding the compound to agar medium [28]. For correct exposure the container must be kept closed so that the compound cannot escape [29]. The material of the container, i.e. plastic or glass, is also important as some compounds, especially hydrophobic compounds, can stick to plastic, leading to reduced exposure levels. For the assessed publications, the material of the container and if it was open or closed was mostly not mentioned but could be inferred from the description, such as 96-well plate or petri dish.

The details of examinations are described in all studies (100% 48/48). However, reproductive toxicity is assessed by different parameters, such as number of eggs, number of hatching eggs, the sum of eggs and larvae, hatching time, number of nematodes surviving to each developmental stage—also, the time when the number of offspring was counted greatly differed between studies. Further standardization may be needed for reproducible DART test results. In addition, it is vital that details of adverse events in both dosed populations and controls, including number of Dauer stage nematodes are reported, as their presence can reduce nematode growth. In addition, population composition in terms of life stages and sex, and the method of measuring these parameters should be reported.

The most complete publications, reported on 26 out of 30 criteria [30] that we formulated, the most incomplete reported only 15 of 30 criteria [31, 32] (Fig. 1C).

A good example of quality reporting is Brunquell *et al.* [33], a study on toxic effect of caffeine and cafestol. In particular, the manufacturer as well as batch number were reported which is specifically important as these compounds can be obtained in different purities and forms. Both small-scale studies investigating only one compound and large-scale studies investigating an array of compounds [34] can be found reporting on the reliability criteria to the same high level, whereas other high-throughput screens do not report on all these criteria [35]. At this point authors are not required to provide these details and can also not be criticized for not doing so. We want to stress that insufficient documentation does not mean poor study design, or poor study quality, but rather prevents taking the study results along in safety assessments [36].

We consider the implementation and reporting of quality criteria and well-defined test methods a critical factor for reliable implementation of NAM test methodologies as alternatives for mammalian testing not only for DART but also for testing other types of toxicity or even health-promoting effects. We provide a framework for relevant and essential *C. elegans* reporting criteria in Table 2 as a step in that direction, for researchers to check if they report essential experimental details, and for regulators to assess study data for inclusion in regulatory hazard assessment of chemicals.

## Conclusion

Literature of the last 2 years shows that the number of studies on DART in *C. elegans* is currently limited. Nevertheless, *C. elegans* studies could provide valuable information on DART, given that studies are carried out according to quality criteria. Here we propose a set of 30 criteria, for which details need to be described in a study about DART in *C. elegans*. We assessed existing literature for current adherence to these criteria. We found that researchers almost always include information about the compounds, such as the manufacturer, although specific details such as batch numbers and purity are mostly missing. The culture conditions are often not described in enough detail to judge if the experimental outcome is reliable and reproducible. Proper testing is essential for the success of NAM to be embraced in the future to ensure reliability. With the provided framework of essential reporting criteria, we aim to advance the reduction, refinement, and replacement of animal testing using *C. elegans* in a reliable manner in the future.

## Supplementary Material

Guideline_Supplementary_information_tfab109
