# Supplementary material for: Towards a reporting guideline for developmental and reproductive toxicology testing in C. elegans and other nematodes
Source: Toxicol Res (Camb). 2021 Nov 28;10(6):1202–10. doi: 10.1093/toxres/tfab109 (PMC8692742; doi:10.1093/toxres/tfab109)
Supplement: Guideline_Supplementary_information_tfab109 [file guideline_supplementary_information_tfab109.docx]

**Supplementary information**

Pubmed Queries to retrieve DART related publications, codes correspond to names of tables in Supplementary Table 1:

**CE200220:**

((("Toxicity Tests"[Mesh] OR "toxicity"[Subheading] OR toxic*[tiab] OR toxin*[tiab])

AND

(development*[tiab] OR reproduct*[tiab])) OR "Teratogens"[Mesh] OR "Teratogenesis"[Mesh] OR "Abnormalities, Drug-Induced"[Mesh] OR teratog*[tiab] OR congenital abnormal* [tiab] OR malform*[tiab] OR embryotoxi*[tiab] OR embryo test*[tiab] OR embryonic test*[tiab] OR maternal exposure [Mesh] OR  maternal exposure* [tiab])

AND ("**Caenorhabditis** elegans"[Mesh] OR Caenorhabditis[tiab] OR C.elegans [tiab])

AND 2018:2020[dp]

**DR200220:**

((("Toxicity Tests"[Mesh] OR "toxicity"[Subheading] OR toxic*[tiab] OR toxin*[tiab])

AND

(development*[tiab] OR reproduct*[tiab])) OR "Teratogens"[Mesh] OR "Teratogenesis"[Mesh] OR "Abnormalities, Drug-Induced"[Mesh] OR teratog*[tiab] OR congenital abnormal* [tiab] OR malform*[tiab] OR embryotoxi*[tiab] OR embryo test*[tiab] OR embryonic test*[tiab] OR maternal exposure [Mesh] OR  maternal exposure* [tiab])

AND  ("**Zebrafish**"[Mesh] OR zebrafish*[tiab] OR zebra fish*[tiab] OR Danio rerio[tiab] OR zebra danio[tiab] OR Brachydanio rerio[tiab])

AND 2018:2020[dp]

**DD200220:**

((("Toxicity Tests"[Mesh] OR "toxicity"[Subheading] OR toxic*[tiab] OR toxin*[tiab])

AND

(development*[tiab] OR reproduct*[tiab])) OR "Teratogens"[Mesh] OR "Teratogenesis"[Mesh] OR "Abnormalities, Drug-Induced"[Mesh] OR teratog*[tiab] OR congenital abnormal* [tiab] OR malform*[tiab] OR embryotoxi*[tiab] OR embryo test*[tiab] OR embryonic test*[tiab] OR maternal exposure [Mesh] OR  maternal exposure* [tiab])

AND  (**Dictyostelium** [mesh] OR Dictyostelium* [tiab] OR discoideum [tiab] OR slime mold* [tiab])

AND 2018:2020[dp]

**MM200220:**

((("Toxicity Tests"[Mesh] OR "toxicity"[Subheading] OR toxic*[tiab] OR toxin*[tiab])

AND

(development*[tiab] OR reproduct*[tiab])) OR "Teratogens"[Mesh] OR "Teratogenesis"[Mesh] OR "Abnormalities, Drug-Induced"[Mesh] OR teratog*[tiab] OR congenital abnormal* [tiab] OR malform*[tiab] OR embryotoxi*[tiab] OR embryo test*[tiab] OR embryonic test*[tiab] OR maternal exposure [Mesh] OR  maternal exposure* [tiab])

AND  (**Mice** [Mesh] OR mice[Tiab] OR mus[Tiab] OR mouse[Tiab] OR murine[Tiab])

AND 2018:2020[dp]

**RN200220:**

((("Toxicity Tests"[Mesh] OR "toxicity"[Subheading] OR toxic*[tiab] OR toxin*[tiab])

AND

(development*[tiab] OR reproduct*[tiab])) OR "Teratogens"[Mesh] OR "Teratogenesis"[Mesh] OR "Abnormalities, Drug-Induced"[Mesh] OR teratog*[tiab] OR congenital abnormal* [tiab] OR malform*[tiab] OR embryotoxi*[tiab] OR embryo test*[tiab] OR embryonic test*[tiab] OR maternal exposure [Mesh] OR  maternal exposure* [tiab])

AND  (**Rats** [Mesh] OR rats[Tiab] OR rat[Tiab] OR rattus [tiab] OR norvegicus [tiab] OR Long-Evans[tiab] OR Wistar [tiab] OR sprague dawley [tiab] OR  lister hooded [tiab])

AND 2018:2020[dp]

**OC200220:**

((("Toxicity Tests"[Mesh] OR "toxicity"[Subheading] OR toxic*[tiab] OR toxin*[tiab])

AND

(development*[tiab] OR reproduct*[tiab])) OR "Teratogens"[Mesh] OR "Teratogenesis"[Mesh] OR "Abnormalities, Drug-Induced"[Mesh] OR teratog*[tiab] OR congenital abnormal* [tiab] OR malform*[tiab] OR embryotoxi*[tiab] OR embryo test*[tiab] OR embryonic test*[tiab] OR maternal exposure [Mesh] OR  maternal exposure* [tiab])

AND  ("**Rabbits**"[Mesh] OR rabbit[tiab] OR rabbits[tiab] OR "Oryctolagus"[tiab])

AND 2018:2020[dp]

**Supplementary Table 1** PubMed results and list of full text publications scored with the 30 criteria
